# New generalized-*X* family: Modeling the reliability engineering applications

**DOI:** 10.1371/journal.pone.0248312

**Published:** 2021-03-31

**Authors:** Wanting Wang, Zubair Ahmad, Omid Kharazmi, Clement Boateng Ampadu, E. H. Hafez, Marwa M. Mohie El-Din

**Affiliations:** 1 College of Finance, Capital University of Economics and Business, Beijing, China; 2 Department of Statistics, Yazd University, Yazd, Iran; 3 Department of Statistics, Faculty of Sciences, Vali-e-Asr University of Rafsanjan, Rafsanjan, Iran; 4 31 Carrolton Road, Boston, MA, United states of America; 5 Department of Mathematics, Faculty of Science, Helwan University, Cairo, Egypt; 6 Department of Mathematical and Natural Sciences, Faculty of Engineering, Egyptian Russian University, Badr, Egypt; Tongii University, CHINA

## Abstract

As is already known, statistical models are very important for modeling data in applied fields, particularly in engineering, medicine, and many other disciplines. In this paper, we propose a new family to introduce new distributions suitable for modeling reliability engineering data. We called our proposed family a new generalized-*X* family of distributions. For the practical illustration, we introduced a new special sub-model, called the new generalized-Weibull distribution, to describe the new family’s significance. For the proposed family, we introduced some mathematical reliability properties. The maximum likelihood estimators for the parameters of the new generalized-X distributions are derived. For assessing the performance of these estimators, a comprehensive Monte Carlo simulation study is carried out. To assess the efficiency of the proposed model, the new generalized-Weibull model is applied to the coating machine failure time data. Finally, Bayesian analysis and performance of Gibbs sampling for the coating machine failure time data are also carried out. Furthermore, the measures such as Gelman-Rubin, Geweke and Raftery-Lewis are used to track algorithm convergence.

## 1 Introduction

Within the area of reliability engineering and other related fields, modeling of data related to lifetime events is very crucial. A range of probability models, such as Weibull, gamma, exponential, etc., are available for modeling lifetime data. However, in many cases, these classical models are not suitable for modeling lifetime data, and there is always a clear need for modified forms of these existing distributions. Therefore, the researchers have introduced new families that are more advantageous than the old ones. For more reading about statistical distributions using different approaches; see [1].

In the literature, the Weibull distribution is the most outstanding one that has broadly been used in reliability engineering and in other various areas of research; see, for instance, [2]. Although the Weibull distribution is often used, the confined structure of its hazard function (hf) can only be decreasing, increasing or constant. Generally, many practical problems require a flexible range of hf, for example, the lifetime events that exhibit a bathtub-shaped hf such as human mortality and life cycles of electronic machines and components. Researchers in the past couple of years developed more flexible extensions of the Weibull model to model reliability data adequately. For the recent survey about such distributions, we refer to [3].

The foremost goal of this research is to introduce a new flexible modification of the Weibull model by means of inducting one additional parameter. The induction of the additional parameter leads to the greater flexibility to enhance goodness-of-fit to reliability data. In fact, we show empirically that the new extension of the Weibull distribution offers the best fit to the coating machine failure time data than the two-parameter, three-parameter, and four-parameter competitive distributions (see section 5). The practical example really shows that the proposed distribution is a good alternative candidate for modeling reliability data.

Now we introduce the proposed family of distributions called a new generalized-*X* (NG-*X*) family. A random variable *X* is said to follow a NG-*X* family, if its cumulative distribution function (cdf) is given by

$$\begin{array}{c} G(x;\theta ,\xi )=1-\{\frac{{\left[1-F(x;\xi )\right]}^{\theta }}{e^{F(x;\xi )}}\},\;\;\;\;\;\;\theta >0,\;x,\xi \in R, \end{array}$$ (1)

where *θ* is an extra shape parameter, and *F*(*x*;*ξ*) is the baseline cdf which may depend on the parameter vector ξ∈R. By adding the additional shape parameter, the NG-*X* distributions can provide best fit to reliability engineering data. The corresponding density function is

$$\begin{array}{c} g(x;\theta ,\xi )=f(x;\xi ){\left[1-F(x;\xi )\right]}^{\theta -1}\{\frac{\left(1+\theta \right)-F(x;\xi )}{e^{F(x;\xi )}}\},\;\;\;\;\;\;x\in R. \end{array}$$ (2)

The reliability function *S*(*x*;*θ*, *ξ*) and the hf *h*(*x*;*θ*, *ξ*) of NG-*X* distributions are given by

$$\begin{array}{c} S(x;\theta ,\xi )=\frac{{\left[1-F(x;\xi )\right]}^{\theta }}{e^{F(x;\xi )}},\;\;\;\;\;\;x>0, \end{array}$$

and

$$\begin{array}{c} h(x;\theta ,\xi )=\frac{f(x;\xi )}{1-F(x;\xi )}[\left(1+\theta \right)-F(x;\xi )],\;\;\;\;\;\;x>0, \end{array}$$

respectively.

Using the NG*-X* distributions approach, we introduce a new form of the Weibull model called, a new generalized Weibull (NG-Weibull) distribution. Furthermore, we consider maximum likelihood (Non-Bayesian) and Bayesian procedures in order to estimate the unknown parameters of the NG-Weibull model. In the Bayesian discussion, we consider different types of symmetric and asymmetric loss functions such as squared error loss, weighted squared error, precautionary, *K*-loss, and modified squared error loss function to estimate the unknown parameters of the NG-Weibull model. Since all the parameters are positive, we use gamma prior distributions. Bayesian 95% credible and highest posterior density (HPD) intervals (see [4]) are obtained for every parameter of the NG-Weibull model. We used the Gibbs sampling technique to get posterior samples. From a graphical point of view, we graphed the posterior density function plots. Next, for evaluating the MCMC procedure in Bayesian analysis, we reported diagnostics measures such as Gelman-Rubin, Geweke, and Raftery-Lewis for checking the convergence of the algorithm.

This paper is outlined in the following manner: In Section 2, we define a NG-*X* family. Some mathematical properties of NG-*X* distributions are derived in Section 3. Section 4 is specified for obtaining the estimates using the maximum likelihood estimation, and the Monte Carlo simulation study is also provided in the same section. Section 5 is concerned with the goodness of fit of the proposed distribution. In this section, we showed that NG-Weibull model provides fit to reliability engineering data. Section 6 offers the Bayesian analysis. The future research directions are provided in Section 7. Some concluding comments are presented in Section 8.

## 2 A new generalized Weibull model

Let $F\left(x;\xi \right)=1-e^{-\gamma x^{\alpha }},\;\;x\geq 0,$ be the cdf of the Weibull distribution, where *ξ* = (*α*, *γ*). Then, a random variable say *X* is said to follow the NG-Weibull distribution, if its cdf is given by

$$\begin{array}{c} G(x;\theta ,\xi )=1-\{\frac{e^{-\theta \gamma x^{\alpha }}}{e^{\left(1-e^{-\gamma x^{\alpha }}\right)}}\},\;\;\;\;\;\;x\geq 0. \end{array}$$ (3)

The density function corresponding to Eq (3) is

$$\begin{array}{c} g(x;\theta ,\xi )=\alpha \gamma x^{\alpha -1}e^{-\theta \gamma x^{\alpha }}\{\frac{\theta +e^{-\gamma x^{\alpha }}}{e^{\left(1-e^{-\gamma x^{\alpha }}\right)}}\},\;\;\;\;\;\;x>0. \end{array}$$ (4)

The reliability function and hf of the NG-Weibull distribution are given by

$$\begin{array}{c} S(x;\theta ,\xi )=\frac{e^{-\theta \gamma x^{\alpha }}}{e^{\left(1-e^{-\gamma x^{\alpha }}\right)}},\;\;\;\;\;\;x>0, \end{array}$$

and

$$\begin{array}{c} h(x;\theta ,\xi )=\alpha \gamma x^{\alpha -1}[\theta +e^{-\gamma x^{\alpha }}],\;\;\;\;\;\;x>0, \end{array}$$

respectively.

**Fig 1:**
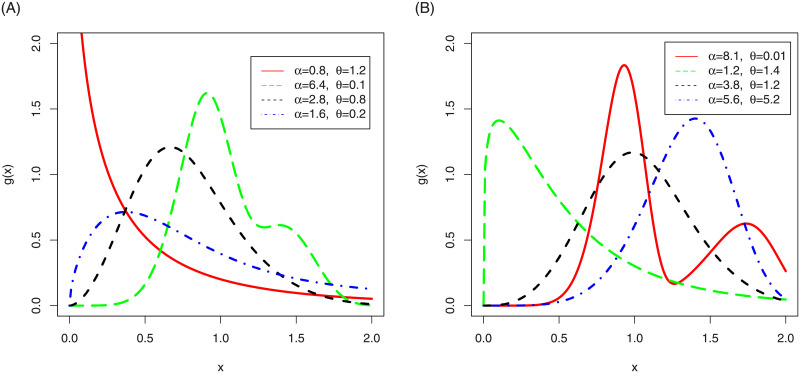
The density plots of the NG-Weibull distribution using *γ* = 1 and different values *α* and *θ*.

**Fig 2:**
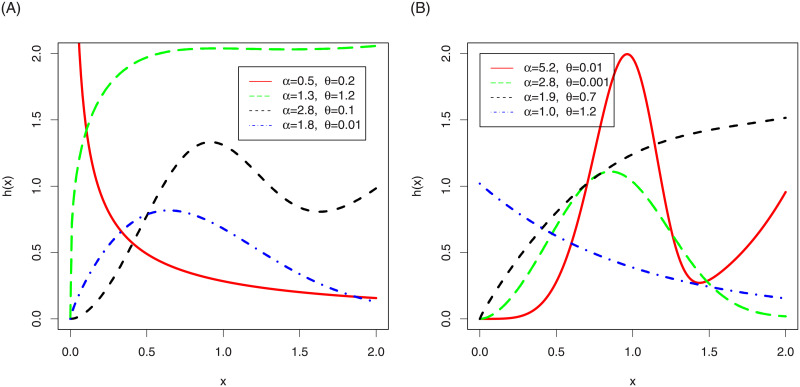
The hf plots of the NG-Weibull distribution using *γ* = 1 and different values *α* and *θ*.

In the Fig 1, we have sketched the density function plots of the NG-Weibull distribution. Fig 1 shows that the NG-Weibull density can be reverse J-shape, symmetric, positively skewed, negatively skewed, and bi-model. The hf plots of the NG-Weibull model are presented in Fig 2. The NG-Weibull hf can be monotonically decreasing, increasing, uni-modal, and modified uni-modal shaped.

## 3 Mathematical properties

In this part of the paper, we derived the mathematical properties associated with the NG-*X* distributions, which include identifiability, quantile function, random number generation, *r*^*th*^ noncentral moments, and the Renyi entropy with numerical illustrations. A characterization theorem extending the NG-*X* class of distributions in terms of the hf is also provided.

### 3.1 Identifiability

The identifiability is an important statistical property that a model must obey to make sure that the inference should be precise. In this subsection, we prove the identifiability property of the NG-*X* distributions. To prove the identifiability property of the NG-*X* distributions, we have to show that *θ*_1_ = *θ*_2_. Let *θ*_1_ and *θ*_2_ be the two parameters having the NG-*X* distributions with cdfs given by *G*(*x*;*θ*_1_, *ξ*) and *G*(*x*;*θ*_2_, *ξ*), respectively. From the definition of identifiability, we have

$$\begin{array}{c} G\left(x;\theta_{1},\xi \right)=G\left(x;\theta_{2},\xi \right), \end{array}$$

$$\begin{array}{c} 1-\{\frac{{\left[1-F(x;\xi )\right]}^{\theta_{1}}}{e^{F(x;\xi )}}\}=1-\{\frac{{\left[1-F(x;\xi )\right]}^{\theta_{2}}}{e^{F(x;\xi )}}\}, \end{array}$$

$$\begin{array}{c} e^{F(x;\xi )}-{\left[1-F(x;\xi )\right]}^{\theta_{1}}=e^{F(x;\xi )}-{\left[1-F(x;\xi )\right]}^{\theta_{2}}, \end{array}$$

$$\begin{array}{c} {\left[1-F(x;\xi )\right]}^{\theta_{1}}={\left[1-F(x;\xi )\right]}^{\theta_{2}}, \end{array}$$

$$\begin{array}{c} \theta_{1}=\theta_{2}. \end{array}$$

### 3.2 Quantile function

**Theorem 1.** *The quantile function of NG-X distributions is given by*

$$\begin{array}{c} Q\left(p\right)=F^{-1}\{1-\theta W(\frac{e^{1/\theta }{\left(1-p\right)}^{1/\theta }}{\theta })\}, \end{array}$$

*where F*^−1^ *is the quantile of the distribution with cdf F*(*x*), 0 < *p* < 1, *and W*(*z*) *gives the principal solution for w* in *z* = *we*^*w*^

**Proof**.

Let 0 < *p* < 1, we must solve the following equation for *Q*(*p*)

$$\begin{array}{c} p=1-\frac{{\left(1-F\left(Q\left(p\right)\right)\right)}^{\theta }}{e^{F(Q(p))}}. \end{array}$$

Let *y* = *F*(*Q*(*p*)). Using software like MATHEMATICA, if we solve the equation below for *y*

$$\begin{array}{c} p=1-\frac{{\left(1-y\right)}^{\theta }}{e^{y}}, \end{array}$$

we get

$$\begin{array}{c} F\left(Q\left(p\right)\right)=1-\theta W(\frac{e^{1/\theta }{\left(1-p\right)}^{1/\theta }}{\theta }). \end{array}$$

Thus

$$Q(p)=F^{-1}\{1-\theta W(\frac{e^{1/\theta }{\left(1-p\right)}^{1/\theta }}{\theta })\}.$$

**Notation:** *If the cdf of the Weibull distribution is given by*

$$\begin{array}{c} F\left(x;a,b\right)=1-e^{-\gamma x^{\alpha }}, \end{array}$$

*we write*

$$\begin{array}{c} Y∼NGW(\alpha ,\theta ,\gamma ), \end{array}$$

*if Y is a NG-Weibull random variable*.

Some numerical values of the quantile measure are provided in Table 1.

**Table 1:** Some quantile values of the NG-Weibull distribution.

*x*	*Q*(*x*) of NG-Weibull(2.5,2,0.5)
0.1	0.0869371
0.2	0.126821
0.3	0.160766
0.4	0.192982
0.5	0.2256
0.6	0.260495
0.7	0.300215
0.8	0.349663
0.9	0.423211

### 3.3 Random number generation

If *U* ∼ Uniform(0, 1), then random numbers from NG-*X* distributions can be obtained from

$$\begin{array}{c} X=F^{-1}\{1-\theta W(\frac{e^{1/\theta }{\left(1-U\right)}^{1/\theta }}{\theta })\}, \end{array}$$

where *F*^−1^ is the quantile of the distribution with cdf *F*(*x*), and *W*(*z*) gives the principal solution for *w* in *z* = *we*^*w*^.

### 3.4 The *r*^*th*^ non central moments

**Theorem 2.** *The r*^*th*^ *Non Central Moments of NG-X distributions can be expressed as*

$$\begin{array}{c} \mu_{r}^{\prime }=\sum_{i=0}^{\infty }\sum_{k=0}^{\infty }\sum_{n=k}^{\infty }\sum_{q=0}^{\infty }\Omega_{i,k,n,q}E\left[U^{q}\right], \end{array}$$

*where* Ω_*i*,*k*, *n*, *q*_ *is defined as in the proof of the theorem, E*[⋅] *denotes an expectation, and U* ∼ *Uniform(0, 1).*

**Proof.** From subsection 3.3, we know if *U* ∼ Uniform(0, 1), then the following random variable

$$\begin{array}{c} X=F^{-1}\{1-\theta W(\frac{e^{1/\theta }{\left(1-U\right)}^{1/\theta }}{\theta })\}, \end{array}$$

where *F*^−1^ is the quantile of the distribution with cdf *F*(*x*), and *W*(*z*) gives the principal solution for *w* in *z* = *we*^*w*^, follows the NG-*X* family of distributions.

According to [5], we can write

$$\begin{array}{c} Q_{X}\left(u\right)=\sum_{i=0}^{\infty }h_{i}u^{i}, \end{array}$$

where the coefficients are suitably chosen real numbers that depend on the parameters of the *F*(*x*) distribution. For a power series raised to a positive integer *r* ≥ 1, we have

$$\begin{array}{c} {\left(Q_{X}\left(u\right)\right)}^{r}={\left(\sum_{i=0}^{\infty }h_{i}u_{i}\right)}^{r}=\sum_{i=0}^{\infty }\delta_{r,i}u^{i}, \end{array}$$

where *δ*_*r*,*i*_ are obtained from $\delta_{r,i}={\left(ih_{0}\right)}^{-1}\sum_{s=1}^{i}\left[s\left(r+1\right)-i\right]h_{s}\delta_{r,i-s}$ with $\delta_{r,0}=h_{0}^{r}$ for *i* = 1, 2, …; see [6]. Thus we have the following

$$\begin{array}{c} \mu_{r}^{\prime }=\sum_{i=0}^{\infty }\delta_{r,i}E[{\left(1-\theta W(\frac{e^{1/\theta }{\left(1-U\right)}^{1/\theta }}{\theta })\right)}^{i}], \end{array}$$

where *E*(⋅) is an expectation. By the binomial series, we can write

$$\begin{array}{c} {\left(1-\theta W(\frac{e^{1/\theta }{\left(1-U\right)}^{1/\theta }}{\theta })\right)}^{i}=\sum_{k=0}^{\infty }(\begin{array}{c} i\\ k \end{array}){\left(-1\right)}^{k}\theta^{k}W{\left(\frac{e^{1/\theta }{\left(1-U\right)}^{1/\theta }}{\theta }\right)}^{k}. \end{array}$$

By using integer powers of the Lambert W function we can write

$$\begin{array}{c} W{\left(\frac{e^{1/\theta }{\left(1-U\right)}^{1/\theta }}{\theta }\right)}^{k}=\sum_{n=k}^{\infty }\frac{-k{\left(-n\right)}^{n-k-1}}{(n-k)!}\;\frac{e^{\frac{n}{\theta }}{\left(1-U\right)}^{\frac{n}{\theta }}}{\theta^{n}}. \end{array}$$

By the binomial series we can write

$$\begin{array}{c} {\left(1-U\right)}^{\frac{n}{\theta }}=\sum_{q=0}^{\infty }(\begin{array}{c} \frac{n}{\theta }\\ q \end{array}){\left(-1\right)}^{q}U^{q}. \end{array}$$

Put

$$\begin{array}{c} \Omega_{i,k,n,q}=\delta_{r,i}(\begin{array}{c} i\\ k \end{array}){\left(-1\right)}^{k}\theta^{k}\frac{-k{\left(-n\right)}^{n-k-1}}{(n-k)!}\frac{e^{\frac{n}{\theta }}}{\theta^{n}}(\begin{array}{c} \frac{n}{\theta }\\ q \end{array}){\left(-1\right)}^{q}. \end{array}$$

It follows that

$$\begin{array}{c} \mu_{r}^{\prime }=\sum_{i=0}^{\infty }\sum_{k=0}^{\infty }\sum_{n=k}^{\infty }\sum_{q=0}^{\infty }\Omega_{i,k,n,q}E\left[U^{q}\right]. \end{array}$$

Some numerical description of the ordinary moments are presented in Table 2.

**Table 2:** Numerical description for some ordinary moments of the NG-Weibull distribution.

*x*	*E*[*X*^*r*^] of NG-Weibull(2.5,2,0.5)
1	0.243489
2	0.0767798
3	0.0289746
4	0.0125534
5	0.00608487
6	0.00324147
7	0.00187295
8	0.00116198
9	0.000767809

### 3.5 Renyi entropy

**Theorem 3.** *The Renyi entropy of the NG-X distributions, for δ* ≠ 1, *δ* > 0, *can be expressed as*

$$\begin{array}{c} I_{R}\left(\delta \right)=\frac{1}{1-\delta }\log(\sum_{k=0}^{\infty }\sum_{q=0}^{\delta }\sum_{r=0}^{\infty }\Omega_{k,q,r}\int_{-\infty }^{\infty }f{\left(x\right)}^{\delta }F{\left(x\right)}^{k+q+r}dx), \end{array}$$

*where X is a random variable with cdf F*(*x*) *and pdf f*(*x*), *and* Ω_*k*,*q*, *r*_ *is defined as in the proof of the theorem.*

**Proof.** Recall the pdf of NG-*X* distributions is given by

$$\begin{array}{c} g\left(x\right)=f\left(x\right){\left(1-F\left(x\right)\right)}^{\theta -1}\left(\left(1+\theta \right)-F\left(x\right)\right)e^{-F(x)}. \end{array}$$

We first find an expansion for *g*(*x*)^*δ*^ where *δ* ≠ 1 and *δ* > 0. By the binomial series we can write

$$\begin{array}{c} {\left(1-F\left(x\right)\right)}^{\delta (\theta -1)}=\sum_{k=0}^{\infty }(\begin{array}{c} \delta (\theta -1)\\ k \end{array}){\left(-1\right)}^{k}F{\left(x\right)}^{k}. \end{array}$$

By the binomial theorem we can write

$$\begin{array}{c} {\left(\left(1+\theta )-F(x\right)\right)}^{\delta }=\sum_{q=0}^{\delta }(\begin{array}{c} q\\ \delta \end{array}){\left(1+\theta \right)}^{\delta -q}{\left(-1\right)}^{q}F{\left(x\right)}^{q}. \end{array}$$

By the power series representation for the exponential function, we can write

$$\begin{array}{c} e^{-\delta F(x)}=\sum_{r=0}^{\infty }\frac{{\left(-1\right)}^{r}\delta^{r}F{\left(x\right)}^{r}}{r!}. \end{array}$$

Put

$$\begin{array}{c} \Omega_{k,q,r}=(\begin{array}{c} \delta (\theta -1)\\ k \end{array}){\left(-1\right)}^{k}(\begin{array}{c} q\\ \delta \end{array}){\left(1+\theta \right)}^{\delta -q}{\left(-1\right)}^{q}\frac{{\left(-1\right)}^{r}\delta^{r}}{r!}. \end{array}$$

It follows that

$$\begin{array}{c} g{\left(x\right)}^{\delta }=\sum_{k=0}^{\infty }\sum_{q=0}^{\delta }\sum_{r=0}^{\infty }\Omega_{k,q,r}f{\left(x\right)}^{\delta }F{\left(x\right)}^{k+q+r}. \end{array}$$

Therefore, the Renyi entropy is

$$\begin{array}{c} I_{R}\left(\delta \right)=\frac{1}{1-\delta }\log(\sum_{k=0}^{\infty }\sum_{q=0}^{\delta }\sum_{r=0}^{\infty }\Omega_{k,q,r}\int_{-\infty }^{\infty }f{\left(x\right)}^{\delta }F{\left(x\right)}^{k+q+r}dx). \end{array}$$

### 3.6 Characterization theorem

It is clear that *hf*, of a function, *F*, that can be differentiated twice satisfies the following differential equation

$$\begin{array}{c} \frac{f^{\prime }\left(x\right)}{f(x)}=\frac{h_{F}^{\prime }\left(x\right)}{h_{F}\left(x\right)}-h_{F}\left(x\right). \end{array}$$

In this section, we present a Kumaraswamy NG-*X* type distribution. The result here is inspired by [7]. First, let us introduce the following.

**Definition:** *We say a random variable X follows a Kumaraswamy*-*G type distribution if its cdf is given by*

$$\begin{array}{c} F\left(x;\xi \right)=1-{\left(1-G\left(x;\xi \right)\right)}^{2}, \end{array}$$

*where G is some baseline distribution*, *x* ∈ *Supp*(*G*), *and ξ is a vector of parameters in the baseline distribution whose support depends on G*.

**Remark:** *Note that if we take* λ = 1 *and φ* = 2 *in* Eq (1) *of* [8], *then we get the cdf in the above definition.*

The pdf of the Kumaraswamy-*G* type distribution is given by

$$\begin{array}{c} f(x;\xi )=2g(x;\xi )(1-G(x;\xi )), \end{array}$$

where *g* is the pdf of the baseline distribution. Clearly the hf of the Kumaraswamy-*G* type distribution is given by

$$\begin{array}{c} h\left(x;\xi \right)=\frac{2g(x;\xi )}{\left(1-G(x;\xi )\right)}. \end{array}$$

**Theorem 4.** *Let* X:Ω↦R *be a continuous random variable*. *The pdf of X is*

$$\begin{array}{c} 2g(x;\xi )(1-G(x;\xi )), \end{array}$$

*for some baseline distribution with pdf g and cdf G*, *if and only if its hazard rate function h*(*x*) *satisfies the following differential equation*

$$\begin{array}{c} h^{\prime }\left(x\right)-\frac{g^{\prime }\left(x\right)}{g(x)}h\left(x\right)=\frac{2g{\left(x\right)}^{2}}{{\left(1-G\left(x\right)\right)}^{2}}, \end{array}$$

*with boundary condition h*(0) = 2*g*(0).

**Proof.** If *X* has pdf as stated in the theorem, then the differential equation as stated holds. Now if the stated differential equation holds, then

$$\begin{array}{c} \frac{d}{dx}\{g{\left(x\right)}^{-1}h\left(x\right)\}=2\frac{d}{dx}\{{\left(1-G\left(x\right)\right)}^{-1}\}, \end{array}$$

which implies

$$\begin{array}{c} h\left(x\right)=\frac{2g(x)}{1-G(x)}, \end{array}$$

which is the hf of the Kumaraswamy-*G* type distribution.

Clearly, a characterization of the Kumaraswamy NG-*X* type distribution is obtained from the above theorem by letting the baseline pdf and cdf given in section 1.

## 4 Maximum likelihood estimation and Monte Carlo simulation

This section is devoted to estimating the NG-*X* parameters using the maximum likelihood estimation approach and providing a comprehensive Monte Carlo (MC) simulation study to assess the maximum likelihood estimators (MLEs) performance.

### 4.1 Maximum likelihood estimation

Let *x*_1_, *x*_2_, …, *x*_*n*_ be the observations of a random sample of size *n* taken from the NG-*X* distribution with the parameter vector Θ = (*θ*, *ξ*)^*T*^. For Θ, the log-likelihood function (LLF) is given by

$$\begin{array}{c} \ell \left(\Theta \right)=\sum_{i=0}^{n}\log f\left(x;\xi \right)+\left(\theta -1\right)\sum_{i=0}^{n}\log\left(1-F\left(x;\xi \right)\right)+\sum_{i=0}^{n}\log\left[\left(\theta +1\right)-F\left(x;\xi \right)\right]-\sum_{i=0}^{n}F\left(x;\xi \right). \end{array}$$ (5)

The partial derivatives of the LLF are given by

$$\begin{array}{c} \frac{\partial }{\partial \theta }\ell \left(\Theta \right)=\sum_{i=0}^{n}\log\left(1-F\left(x;\xi \right)\right)+\sum_{i=0}^{n}\frac{1}{\left(\theta +1)-F(x;\xi \right)}\;, \end{array}$$

and

$$\begin{array}{c} \frac{\partial }{\partial \xi }\ell \left(\Theta \right)=\sum_{i=0}^{n}\frac{1}{f(x;\xi )}-\left(\theta -1\right)\sum_{i=0}^{n}\frac{\partial F(x;\xi )/\partial \xi }{\left[1-F(x;\xi )\right]}-\frac{\partial F(x;\xi )/\partial \xi }{\left[(\theta +1)-F(x;\xi )\right]}-\sum_{i=0}^{n}\partial F\left(x;\xi \right)/\partial \xi \;, \end{array}$$

respectively.

The MLEs of the unknown parameters *θ* and *ξ* of the NG-*X* distributions can be obtained by maximizing $\frac{\partial }{\partial \theta }\ell \left(\Theta \right)=0$ and $\frac{\partial }{\partial \xi }\ell \left(\Theta \right)=0$, respectively.

### 4.2 Monte Carlo simulation study

**Fig 3:**
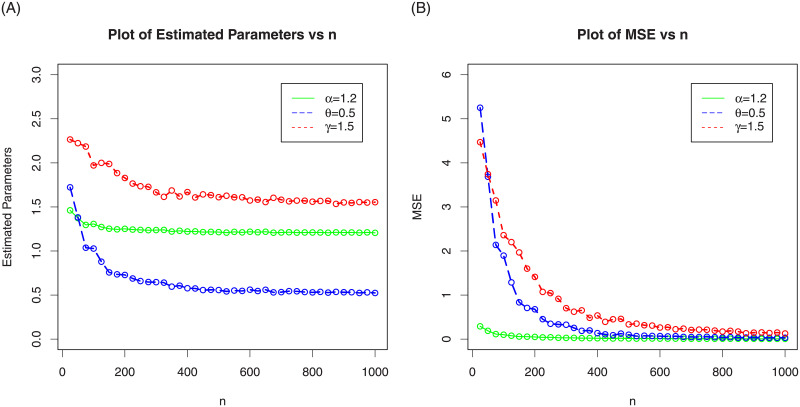
The above graph plots the MLEs and MSEs of the NG-Weibull with values of the parameters given as *α* = 1.2, *θ* = 0.5 and *γ* = 1.5.

**Fig 4:**
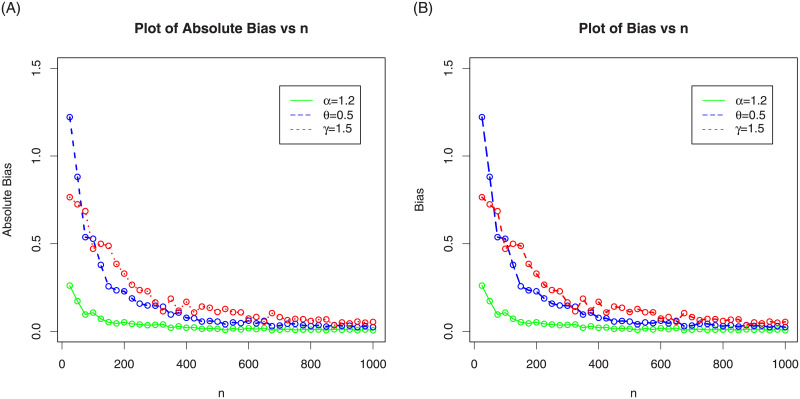
The above graph plots the biases and absolute biases of the NG-Weibull with values of the parameters given as *α* = 1.2, *θ* = 0.5 and *γ* = 1.5.

**Fig 5:**
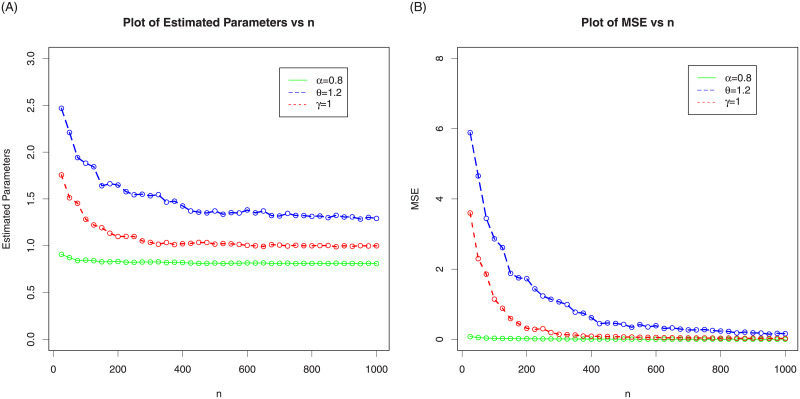
In the above graph we plotted the MLEs and MSEs of the NG-Weibull using parameter values *α* = 0.8, *θ* = 1.2 and *γ* = 1.

**Fig 6:**
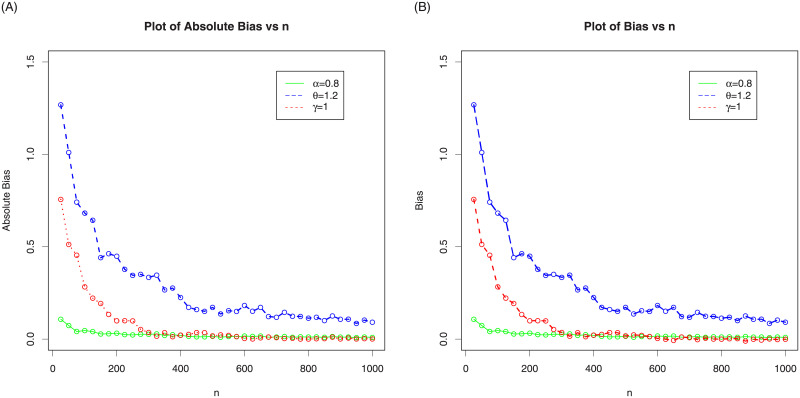
In the above graph we plotted the biases and absolute biases of the NG-Weibull using parameter values *α* = 0.8, *θ* = 1.2 and *γ* = 1.

In the following sub-section, we assess the behavior of the MLEs of NG-Weibull distribution by means of the MC simulation study. The process is carried out by maximizing the LLF using the optim() R-function with the argument method = “L-BFGS-B”. We made 1000 MC-iterations using different sizes of the samples as follows, *n* = 25, 50,…,1000. We computed the average MLEs, the associated mean square errors (MSE), biases and absolute biases. For the first set of MC simulation results, the plots MLEs and MSE are provided in Fig 3 and the plots biases and absolute biases are provided in Fig 4. Whereas, for the second set of MC simulation results, the plots MLEs and MSE are provided in Fig 5 and the plots biases and absolute biases are provided in Fig 6.

## 5 Application on our model from reliability engineering

In this section, we evaluate the usefulness of the NG-Weibull distribution by means of analyzing reliability engineering data taken from [9]. The data represents the failure time of the coating machine given by: 1.00, 1.00, 5.00, 5.50, 12.50, 16.75, 17.75, 20.75, 22.50, 22.75, 25.00, 25.00, 27.25, 30.25, 43.75, 45.00, 48.00, 48.25, 97.50, 99.75, 136.75, 143.50, 207.75, 215.00, 225.50, 235.00, 283.50, 567.00, 970.50. The NG-Weibull is applied to this data, and the comparison of its goodness of fit is made with the other prominent distributions such as Beta Weibull (BW) [10], Kumaraswamy Weibull (Ku-W) distribution [11], Extended Alpha Power Transformed Weibull (Ex-APTW) [12] and type-I heavy-tailed Weibull (TI-HTW) distribution [13].

To figure out about the goodness of fit amongst the competitive distributions, we consider certain goodness of fit measures such as Cramer-Von-Mises (CM) statistic, Anderson Darling (AD) statistic, and Kolmogorov-Smirnov (KS)statistics alongside with its p-values.

Corresponding to the coating machine failure time data, the model parameters’ estimated values with standard errors in the parenthesis are presented in Table 3. The goodness of fit measures of the competitive models are provided in Table 4. From the results reported in Table 4, we can see that the proposed model has lower values of the goodness of fit measures and a high p-value indicating the best fit for the reliability data.

**Table 3:** MLEs of the competing distributions for the coating machine failure time data.

Models	*α*	*γ*	*θ*	*α*_1_	*a*	*b*
NG-Weibull	0.964 (0.2287)	0.018 (0.0105)	0.243 (0.2707)			
TI-HTW	0.525 (0.0746)	0.843 (0.5898)	0.149 (0.1097)			
Ex-APTW	0.510 (0.5094)	0.172 (0.6258)		5.425 (7.0766)		
Ku-W	0.620 (0.3093)	0.501 (1.0970)			0.702 (3.2715)	0.118 (2.0964)
BW	0.478 (0.2696)	0.502 (0.5522)			2.797 (3.1595)	0.344 (0.6646)

**Table 4:** Goodness of fit measures of the competing models for the coating machine failure time data.

Models	CM	AD	KS	p-value
NG-Weibull	0.054	0.328	0.106	0.897
TI-HTW	0.060	0.336	0.124	0.756
Ex-APTW	0.093	0.491	0.142	0.598
Ku-W	0.091	0.546	0.146	0.488
BW	NaN	NaN	0.144	0.603

**Fig 7:**
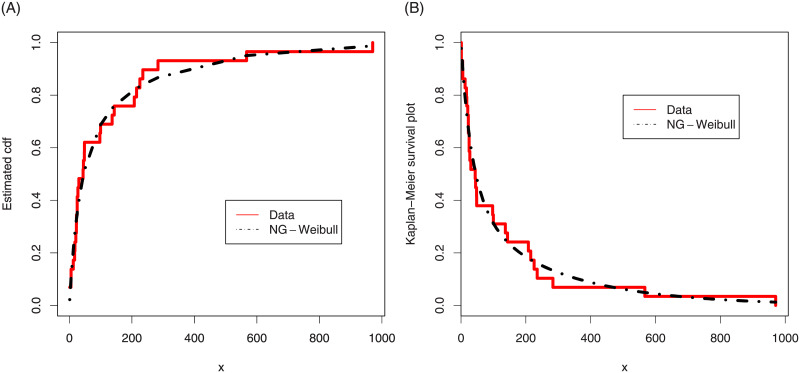
The estimated cdf and Kaplan-Meier survival plots of the NG-Weibull distribution for the coating machine failure time data.

**Fig 8:**
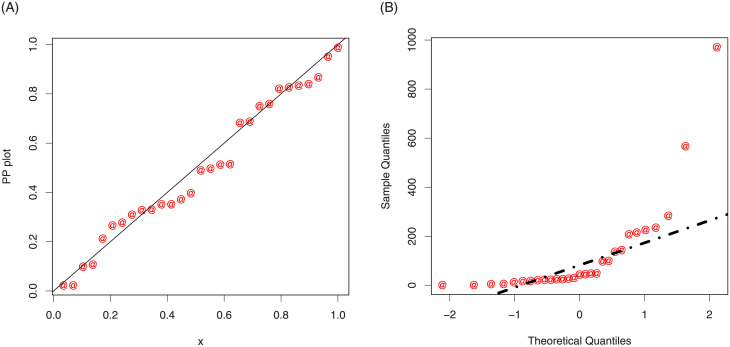
The PP and *QQ* plots of the NG-Weibull distribution for the coating machine failure time data.

In support of the goodness of fit measures given in Table 4, the estimated cdf and Kaplan-Meier survival plot of the NG-Weibull distribution are plotted in Fig 7. The probability-probability (PP) and quantile-quantile (*QQ*) plots are presented in Fig 8. These figures confirm the best fitting of the NG-Weibull to the coating machine failure time data.

Furthermore, for the coating machine failure time data, we calculated the KS statistic values of the NG-Weibull and other considered models. Then, we utilized the parametric bootstrap approach [14], and bootstrapped the p-value for all models. The KS statistic and the corresponding bootstrapped p-value are reported in Table 5. According to the results provided in Table 5, we observe that the NG-Weibull is a good candidate model amongst the competing distributions for modeling engineering reliability data.

**Table 5:** The KS and the corresponding bootstrapped p-value of the fitted models for the coating machine failure time data.

Models	KS	Bootstrapped p-value
NG-Weibull	0.164	0.936
TI-HTW	0.226	0.796
Ex-APTW	0.290	0.619
Ku-W	0.418	0.560
BW	0.347	0.670

## 6 Bayesian estimation

In this section, we consider different types of symmetric and asymmetric loss functions such as squared error loss function (SELF), weighted squared error loss function (WSELF), modified squared error loss function (MSELF), precautionary loss function (PLF) and K-loss function (KLF). These functions associated with Bayesian estimators and posterior risks are reported in Table 6.

**Table 6:** Bayes estimator and posterior risk under different loss functions.

Loss function *L*(*ψ*, *δ*)	Bayes estimator *ψ*_*B*_	Posterior risk *ρ*_*ψ*_
*SELF* = (*ψ* − *d*)^2^	*E*(*ψ*|*x*)	*Var*(*ψ*|*x*)
$WSELF=\frac{{\left(\psi -d\right)}^{2}}{\psi }$	(*E*(*ψ*^−1^|*x*))^−1^	*E*(*ψ*|*x*) − (*E*(*ψ*^−1^|*x*))^−1^
$MSELF={\left(1-\frac{d}{\psi }\right)}^{2}$	$\frac{E(\psi^{-1}|x)}{E(\psi^{-2}|x)}$	$1-\frac{E{\left(\psi^{-1}|x\right)}^{2}}{E(\psi^{-2}|x)}$
$PLF=\frac{{\left(\psi -d\right)}^{2}}{d}$	$\sqrt{E(\psi^{2}|x)}$	$2(\sqrt{E(\psi^{2}|x)}-E\left(\psi |x\right))$
$KLF=(\sqrt{\frac{d}{\psi }-\sqrt{\frac{\psi }{d}}})$	$\sqrt{\frac{E(\psi |x)}{E(\psi^{-1}|x)}}$	$2(\sqrt{E\left(\psi |x\right)E\left(\psi^{-1}|x\right)}-1)$

For more detail, we refer to [15–18]. Next, we provide a Bayesian estimation approach for estimating the parameters of NG-Weibull distribution via analyzing complete sample data.

### 6.1 Joint posterior and marginal posterior distributions

Assume that the parameters *α*, *γ* and *θ* of NG-W distribution have independent prior distributions as

$$\begin{array}{c} \alpha ∼Gamma\left(\alpha_{0},\alpha_{1}\right),\gamma ∼Gamma\left(\gamma_{0},\gamma_{1}\right),\theta ∼Gamma\left(\theta_{0},\theta_{1}\right), \end{array}$$

where the all hyper-parameters *α*_0_, *α*_1_, *γ*_0_, *γ*_1_, *θ*_0_ and *θ*_1_ are positive. Consequently, the joint prior density function is formulated as follows:

$$\begin{array}{ccc} \pi (\alpha ,\gamma ,\theta ) & = & \frac{\alpha_{1}^{\alpha_{0}}\gamma_{1}^{\gamma_{0}}\theta_{1}^{\theta_{0}}}{\Gamma \left(\alpha_{0}\right)\Gamma \left(\gamma_{0}\right)\Gamma \left(\theta_{0}\right)}\alpha^{\alpha_{0}-1}\gamma^{\gamma_{0}-1}\theta^{\theta_{0}}e^{-(\alpha_{1}\alpha +\gamma_{1}\gamma +\theta_{1}\theta )}. \end{array}$$ (6)

For simplicity, let us define the function *ζ* as

$$\begin{array}{c} \zeta \left(\alpha ,\gamma ,\theta \right)=\alpha^{\alpha_{0}-1}\gamma^{\gamma_{0}-1}\theta^{\theta_{0}}e^{-(\alpha_{1}\alpha +\gamma_{1}\gamma +\theta_{1}\theta )},\;\alpha >0,\;\gamma >0,\;\theta >0. \end{array}$$

The joint posterior distribution defined from Eq (6) and the likelihood function *L*(*data*) is

$$\begin{array}{c} \pi^{*}\left(\alpha ,\gamma ,\theta |data\right)\propto \pi \left(\alpha ,\gamma ,\theta \right)L\left(data\right). \end{array}$$ (7)

Therefore, the joint posterior density function can be expressed by

$$\begin{array}{ccc} \pi^{*}\left(\alpha ,\gamma ,\theta |\underline{x}\right) & = & K\zeta \left(\alpha ,\gamma ,\theta \right)L\left(\underline{x},\Psi \right), \end{array}$$ (8)

where

$$\begin{array}{c} L\left(\underline{x};\Psi \right)=\prod_{i=1}^{n}\alpha \gamma x_{i}^{\alpha -1}e^{-\theta \gamma x_{i}^{\alpha }}\frac{\theta +e^{-\gamma x_{i}^{\alpha }}}{e^{1-e^{-\gamma x_{i}^{\alpha }}}}, \end{array}$$ (9)

Ψ = (*α*, *γ*, *θ*) and *K* is given as

$$\begin{array}{ccc} K^{-1} & = & \int_{0}^{\infty }\int_{0}^{\infty }\int_{0}^{\infty }\zeta \left(\alpha ,\gamma ,\theta \right)L\left(\underline{x},\Psi \right)\partial \alpha \partial \gamma \partial \theta . \end{array}$$

Moreover, the marginal posterior density functions of *α*,*γ* and *θ* assuming that Ψ = (Ψ_1_, Ψ_2_, Ψ_3_) = (*α*, *γ*, *θ*), can be given by

$$\begin{array}{c} \pi \left(\Psi_{i}|\underline{x}\right)=\int_{0}^{\infty }\int_{0}^{\infty }\pi^{*}\left(\Psi |\underline{x}\right)\partial \Psi_{j}\partial \Psi_{k}, \end{array}$$ (10)

where *i*, *j*, *k* = 1, 2, 3,*i* ≠ *j* ≠ *k* and Ψ_*i*_ is the *i*^*th*^ member of a vector Ψ.

### 6.2 Bayesian point estimation

Under the marginal posterior density function as in Eq (10) and the loss functions which are given in Table 6. The Bayesian point estimation for the parameter vector Ψ = (Ψ_1_, Ψ_2_, Ψ_3_) = (*α*, *γ*, *θ*) is obtained via minimizing the expectation of loss function under the marginal posterior density as follows:

$$\begin{array}{c} \arg\min_{\delta }\int_{0}^{\infty }L\left(\Psi_{i},\delta \right)\pi \left(\Psi_{j}|\underline{x}\right)\partial \Psi_{i}. \end{array}$$ (11)

However, in practice, because of the intractable integral in relation Eq (11), it is suggested to use the well-known Gibbs sampler [19], or Metropolis Hastings algorithms to generate posterior samples [20]. We will argue about this issue more precisely in subsection 6.5.

### 6.3 Credibility interval

In the Bayesian framework, interval estimation can be done via credibility interval conception. Consider the parameter vector Ψ = (Ψ_1_, Ψ_2_, Ψ_3_) = (*α*, *γ*, *θ*), which is associated with the NG-Weibull distribution and $\pi (\Psi_{i}|\underline{x})$ denote the marginal posterior pdf of the parameter Ψ_*j*_; (*j* = 1, 2, 3) as in Eq (10). For a given value of *η* ∈ (0, 1), the (1 − *η*)100% credibility interval $CI(L_{\Psi_{j}},U_{\Psi_{j}})$ is defined as

$$\begin{array}{c} \;\;\;\;\;\;\;\;\;\;\;\;\;\;\;\;\;\;\;\;\int_{L_{\Psi_{j}}}^{\infty }\pi \left(\Psi_{j}|\underline{x}\right)\partial \Psi_{j}=1-\frac{\eta }{2},\;\;\int_{U_{\Psi_{j}}}^{\infty }\pi \left(\Psi_{j}|\underline{x}\right)\partial \Psi_{i}=\frac{\eta }{2}. \end{array}$$ (12)

By considering the relation Eq (12), it is very difficult to obtain the marginal density from the joint posterior density. We use the Gibbs sampler to generate posterior samples. Let Ψ^1^, …, Ψ^*k*^ (where $\Psi^{i}=\left(\Psi_{1}^{i},\Psi_{2}^{i},\Psi_{3}^{i}\right)$) be a posterior random sample of size *k*, which is extracted from the joint posterior density as in Eq (8). Using these generated posterior samples, the marginal posteriors densities of Ψ_*j*_ given $\underline{x}$ can be given by

$$\begin{array}{c} \;\;\;\;\;\;\;\;\;\;\;\;\;\;\;\;\;\;\;\;\;\frac{1}{K}\sum_{i=1}^{K}\pi^{*}\left(\Psi_{j},\Psi_{-j}^{i}|\underline{x}\right);\;j=1,2,3, \end{array}$$ (13)

where the $\Psi_{-j}^{i}$ shows the vector of posterior samples when *jth* component is removed. Using Eq (13) in Eq (12), one can be able to compute the credibility intervals for Ψ_*j*_, *j* = 1, 2, 3 as follows

$$\begin{array}{c} \frac{1}{K}\sum_{i=1}^{K}\int_{L_{\Psi_{j}}}^{\infty }\pi^{*}(\Psi_{j},\Psi_{-j}^{i}|\underline{x})\partial \Psi_{j}=1-\frac{\eta }{2},\;\;\frac{1}{K}\sum_{i=1}^{K}\int_{U_{\Psi_{j}}}^{\infty }\pi^{*}(\Psi_{j},\Psi_{-j}^{i}|\underline{x})\partial \Psi_{j}=\frac{\eta }{2}. \end{array}$$ (14)

### 6.4 Highest posterior density interval

The HPD interval is a kind of credibility interval with a specific restriction. The (1 − *η*)100% (*i* = 1, …, *p*) HPD interval for Ψ_*j*_, *j* = 1, 2, 3 is the simultaneous solution of the following integral equations

$$\frac{1}{K}\sum_{i=1}^{K}\int_{L_{\Psi_{j}}}^{U_{\Psi_{j}}}\pi^{*}(\Psi_{j},\Psi_{-j}^{i}|\underline{x})\partial \Psi_{j}=1-\eta ,\;\sum_{i=1}^{K}\pi^{*}(L_{\Psi_{j}},\Psi_{-j}^{i}|\underline{x})=\sum_{i=1}^{K}\pi^{*}(U_{\Psi_{j}},\Psi_{-j}^{i}|\underline{x}).$$

### 6.5 Generating posterior samples

It is clear from Eqs (8) and (12) that there is no closed-form for point estimators using different loss functions, because of intractable integrals. So we will try to solve those integrals numerically using MCMC methods. There are a lot of possible methods. One of these methods is known as the Metropolis-Hastings algorithm. Another method for approximation of unsolvable integrals is known as the Gibbs sampling. Suppose that the general model $f(\underline{x}|\psi )$ is associated with the parameter vector ***ψ*** = (*ψ*_1_, *ψ*_2_, …, *ψ*_*p*_) and observed data $\underline{x}$. Thus, the joint posterior distribution is $\pi (\psi_{1},\psi_{2},\ldots ,\psi_{p}|\underline{x})$. We also assume that $\psi_{0}=\left(\psi_{1}^{\left(0\right)},\psi_{2}^{\left(0\right)},\ldots ,\psi_{p}^{\left(0\right)}\right)$ is the initial vector to start the Gibbs sampler. The steps for any iteration, say iteration *k*, are as follows:

- Starting with an initial estimate $\left(\psi_{1}^{\left(0\right)},\psi_{2}^{\left(0\right)},\ldots ,\psi_{p}^{\left(0\right)}\right)$
- Draw $\psi_{1}^{k}$ from $\pi (\psi_{1}|\psi_{2}^{k-1},\psi_{3}^{k-1},\ldots ,\psi_{p}^{k-1},\underline{x})$
- Draw $\psi_{2}^{k}$ from $\pi (\psi_{2}|\psi_{1}^{k},\psi_{3}^{k-1},\ldots ,\psi_{p}^{k-1},\underline{x})$; and so on down to
- Draw $\psi_{p}^{k}$ from $\pi (\psi_{p}|\psi_{1}^{k},\psi_{2}^{k},\ldots ,\psi_{p-1}^{k},\underline{x}).$

In case of the NG-Weibull distribution, by considering the parameter vector Ψ = (*α*, *γ*, *θ*) and initial parameter vector Ψ_0_ = *c*(*α*^0^, *γ*^0^, *θ*^0^), the posterior samples are extracted by above Gibbs sampler where the full conditional distributions are given as

$$\begin{array}{c} \pi (\alpha |\gamma^{k-1},\theta^{k-1},\underline{x})\propto \alpha^{\alpha_{0}+n-1}e^{-\alpha_{1}\alpha }\prod_{i=1}^{n}x_{i}^{\alpha -1}e^{-\theta \gamma x_{i}^{\alpha }}\{\frac{\theta +e^{-\gamma x_{i}^{\alpha }}}{e^{1-e^{-\gamma x_{i}^{\alpha }}}}\}, \end{array}$$ (15)

$$\begin{array}{c} \pi (\gamma |\alpha^{k-1},\theta^{k-1},\underline{x})\propto \gamma^{\gamma_{0}+n-1}e^{-\gamma_{1}\gamma }\prod_{i=1}^{n}e^{-\theta \gamma x_{i}^{\alpha }}\{\frac{\theta +e^{-\gamma x_{i}^{\alpha }}}{e^{1-e^{-\gamma x_{i}^{\alpha }}}}\}, \end{array}$$ (16)

and

$$\begin{array}{c} \pi (\theta |\alpha^{k-1},\gamma^{k-1},\underline{x})\propto \theta^{\theta_{0}-n}e^{-\theta_{1}\theta }\prod_{i=1}^{n}e^{-\theta \gamma x_{i}^{\alpha }}\{\theta +e^{-\gamma x_{i}^{\alpha }}\}. \end{array}$$ (17)

Gibbs sampling processes can be carried out via OpenBUGS software, which is an available version of WinBUGS. Here, since there aren’t any prior information about hyper-parameters in Eq (6), we implement the idea of [21], and the hyper-parameters values are setted as *α*_0_ = *α*_1_ = *γ*_0_ = *γ*_1_ = *θ*_0_ = *θ*_1_ = 0.0001. We can use the MCMC procedure to extract posterior samples of Eq (8) by means of the Gibbs sampling process in OpenBUGS software.

**Fig 9:**
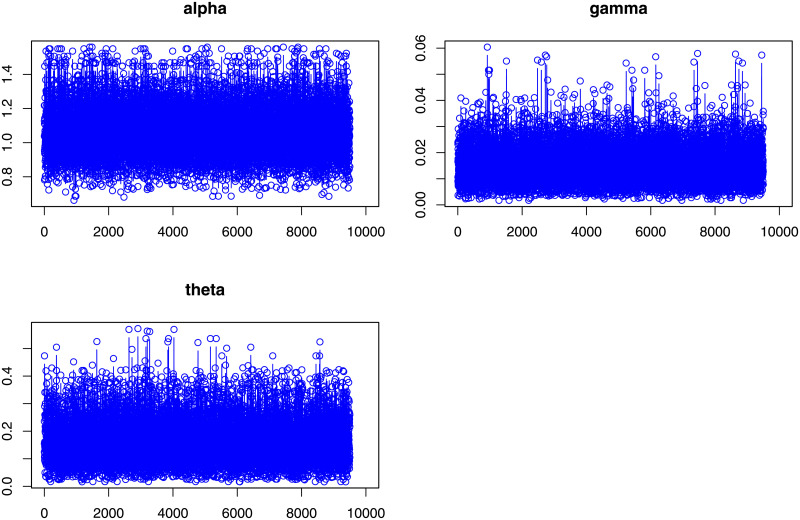
Posterior summary plots of Gibbs sampling performance for the coating machine failure time data (Trace plots).

**Fig 10:**
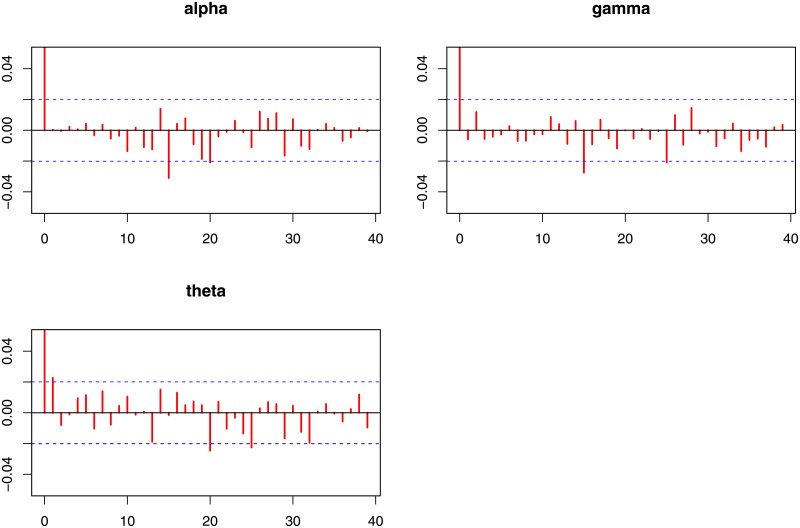
Posterior summary plots of Gibbs sampling performance for the coating machine failure time data (Autocorrelation plots).

**Fig 11:**
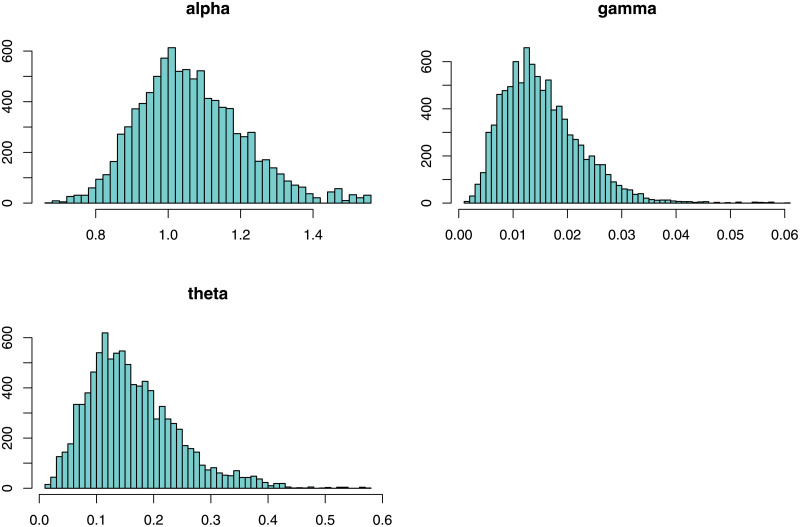
Posterior summary plots of Gibbs sampling performance for the coating machine failure time data (Histogram plots).

Next, we provide Bayesian estimation results. It is evident from equation Eq (10), there are no closed-form expressions for Bayesian estimators, which are extracted based on the loss functions in Table 6. Therefore, an MCMC procedure via the Gibbs sampler process is designed using the expressions provided in Eqs (15), (16) and (17), with 10,000 replicates to obtain the Bayesian estimators. In Table 7, we provide the corresponding point and posterior risk estimations. Furtherer, 95% credible, and HPD intervals are provided in Table 8. In order to provide a visual inspection, the posterior plots such as trace plots are provided in Fig 9, autocorrelation plots are presented in Fig 10, and the histogram plots are sketched in Fig 11. These plots verify that the convergence of Gibbs sampling process has occurred.

**Table 7:** Summary of Bayesian estimation (point estimation and risk) for the coating machine failure time data.

Data	Coating machine failure time data					
Bayes	$\hat{\alpha }\;\left(r_{\hat{\alpha }}\right)$		$\hat{\gamma }\;\left(r_{\hat{\gamma }}\right)$			$\hat{\theta }\;\left(r_{\hat{\theta }}\right)$
Loss functions	Estimate	Risk	Estimate	Risk	Estimate	Risk
SELF	1.0641	0.0211	0.0153	5.1e-04	0.1631	0.0062
WSELF	1.0449	0.0191	0.0121	0.0032	0.1245	0.0386
MSELF	1.0449	0.0177	0.0090	0.2555	0.0860	0.3088
PLF	1.0264	0.0198	0.0169	0.0032	0.1812	0.0361
KLF	1.0739	0.0182	0.0136	0.2538	0.1425	0.2895

**Table 8:** HPD and Credible intervals for the coating machine failure time data.

Parameters	Credible interval	HPD interval
*α*	(0.9624, 1.1560)	(0.8114, 1.3690)
*γ*	(0.0102, 0.0193)	(0.0036, 0.0289)
*θ*	(0.1071, 0.2076)	(0.0322, 0.3254)

**Fig 12:**
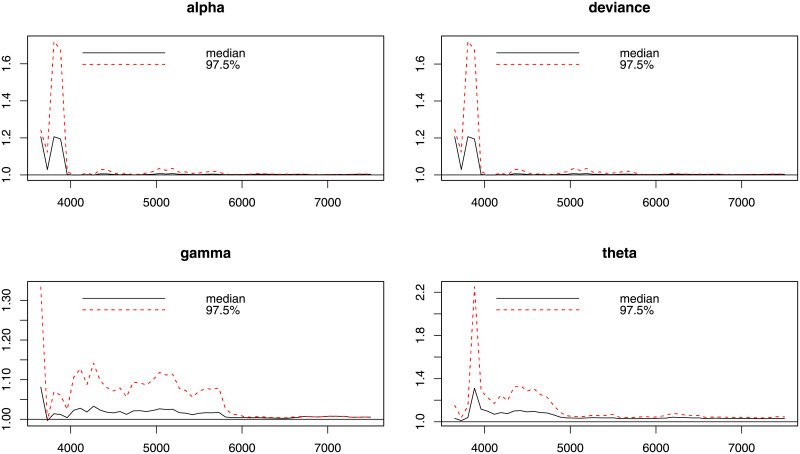
Gelman diagnostic plot for the each parameter of the NG-Weibull distribution using the coating machine failure time data.

**Fig 13:**
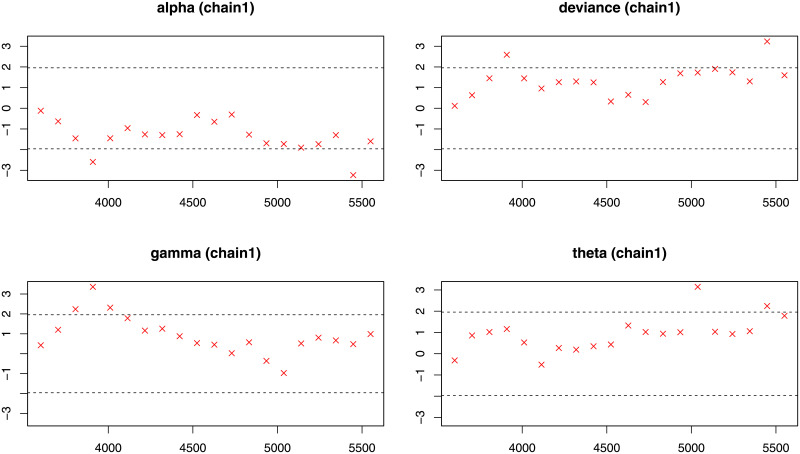
Geweke diagnostic plot (chain1) for the each parameter of the NG-Weibull distribution using the coating machine failure time data.

**Fig 14:**
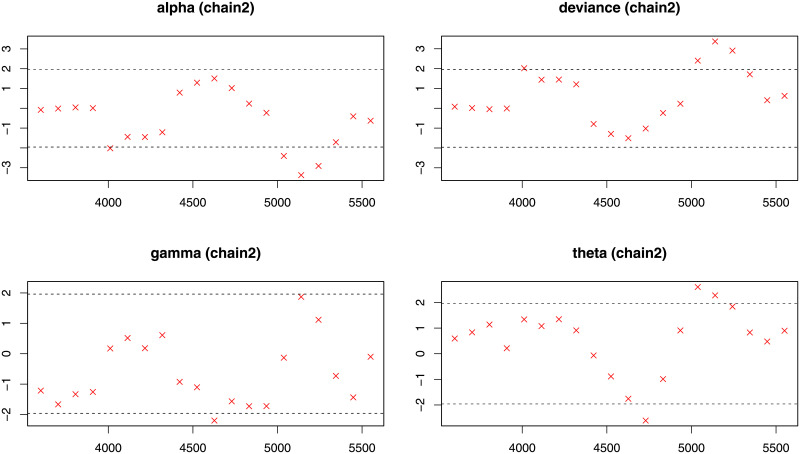
Geweke diagnostic plot (chain2) for the each parameter of the NG-Weibull distribution using the coating machine failure time data.

Next, for evaluation the MCMC procedure in Bayesian analysis, we report some diagnostics measures such as Gelman-Rubin (GR), Geweke (G) and Raftery-Lewis (RL) for checking the convergence of the Gibbs algorithm are provided in Table 9. For more details about these indexes; see [22]. The GR diagnostic for parameters *α*, *γ*, and *θ* is equal to 1. Hence, based on the GR diagnostic measure, the chains are acceptable. Fig 12 shows that the estimates come from state spaces of the corresponding parameters. From Table 9, Geweke’s test statistics for parameters *α*, *γ* and *θ*, are −0.0795, 0.0807 and 0.59370, respectively. Hence, the G diagnostic measure also confirms the acceptance of chains as shown in Figs 13 and 14. Moreover, the reported diagnostic statistics for parameters *α*, *γ* and *θ* based on the RL method do not show a significant degree of dependence between estimates.

**Table 9:** Diagnoses by Gelman-Rubin, Geweke and Raftery-Lewis methods for the parameters *α*, *γ* and *θ* based on the coating machine failure time data.

Parameter	GR	G(*Z*_0.025_ = ±1.96)	RL
*α*	1	-0.0795	4.80
*γ*	1	0.0807	4.79
*θ*	1	0.5937	4.33

## 7 Discussion and future framework

The statistical decision theory helps to address the state of uncertainty and provides potentially a sound framework for dealing with problems of bio-medical, reliability, actuarial, economic and financial decision-making. Among the applied fields, the reliability engineering has received a serious consideration. In the field of reliability theory, modeling of lifetime data is very crucial. The data related to the lifetime of electronic product or any entity, etc., are usually positive and skewed to the right. In lifetime analysis and reliability theory, the failure rate function (also known as hazard rate function) is a prominent reliability characteristic. Among the possible failure rate functions, the unimodal, modified unimodal or bathtub-shaped failure rate curves are well-known in reliability literature. The classical models are not so flexible to model such complex forms of data.

Due to the importance and application of the statistical models in reliability, medical and financial sciences, a reasonable work has been done in the literature aiming to improve the characteristics of the classical models. Although the new improvement has achieved the respective goal, unfortunately, the numbers of parameters have been increased, and the estimation of parameters, statistical inference and derivation of mathematical problems become complicated.

To provide a better description and best fitting to the reliability data, therefore, in the present study, a new family of distributions has been studied. The key goal of introducing the class of distributions is to improve the characteristics of the classical distributions.

From the above theory and discussion, it is quite clear that the researchers are always in search of new flexible distributions. Therefore, to introduce new flexible distributions and bring further flexibility in the model proposed in this paper, we suggest to introduce modified forms of the proposed model. As a future research direction, we suggest to introduce new models useful for modeling data in reliability engineering and financial sciences.

As we stated above that the statistical models with bathtub-shaped failure rate function are very useful in reliability engineering. Here, we suggest a new modification of the proposed distribution to model lifetime data with a bathtub-shaped failure function.

Let *F*(*x*;*ξ*) = 1 − *e*^−*γx*^*α*^ − *ηx*^, be the cdf of the three-parameter modified Weibull (MW) distribution, where *ξ* = (*α*, *γ*, *η*). Then, a random variable say *X* is said to follow the new generalized modified Weibull (NGM-Weibull) distribution, if its cdf is given by

$$\begin{array}{c} G(x;\theta ,\xi )=1-\{\frac{e^{-\theta \gamma x^{\alpha }-\theta \eta x}}{e^{\left(1-e^{-}{{}^{\gamma x^{\alpha }}}^{-\eta x}\right)}}\},\;\;\;\;\;\;x\geq 0. \end{array}$$

The corresponding pdf and hf are given by

$$\begin{array}{c} g(x;\theta ,\xi )=(\alpha \gamma x^{\alpha -1}+\eta )e^{-\theta \gamma x^{\alpha }-\theta \eta x}\{\frac{\theta +e^{-\gamma x^{\alpha }-\eta x}}{e^{\left(1-e^{-}{{}^{\gamma x^{\alpha }}}^{-\eta x}\right)}}\},\;\;\;\;\;\;x>0, \end{array}$$

and

$$\begin{array}{c} h(x;\theta ,\xi )=(\alpha \gamma x^{\alpha -1}+\eta )[\theta +e^{-\gamma x^{\alpha }-\eta x}],\;\;\;\;\;\;x>0, \end{array}$$

respectively.

**Fig 15:**
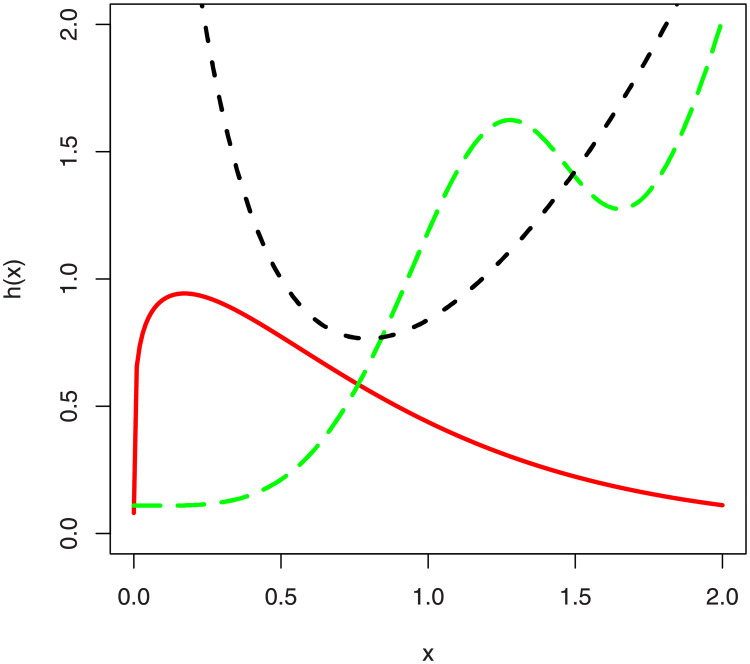
The hf plots of the NGM-Weibull distribution for different values of the parameters.

Different plots for the failure rate function of the NGM-Weibull distribution are provided in Fig 15.

From the plots provided in Fig 15, we can see that the hf of the NGM-Weibull distribution is (i) unimodal for *α* = 1.2, *γ* = 1.2, *θ* = 0.01, *η* = 0.08 (red line), (ii) modified unimodal for *α* = 4.8, *γ* = 0.3, *θ* = 0.1, *η* = 0.1 (green line), and (iii) bathtub-shaped for *α* = 3.5, *γ* = 1, *θ* = 0.1, *η* = 4.6 (black line). Since, the NGM-Weibull captures all the important forms of the hf, therefore, the NGM-Weibull distribution can be a good candidate distribution to model complex form of the reliability engineering data. In the future, therefore, we are intended to model lifetime and reliability data having unimodal, modified unimodal and bathtub-shaped failure rate behavior.

Furthermore, in the practice of actuarial and financial sciences, the heavy-tailed distributions are useful for modeling heavy-tailed financial data sets. Heavy-tailed distributions are those, whose right tail probabilities are heavier than the exponential distribution, and satisfies

$$\begin{array}{c} \lim_{x\to \infty }e^{px}[1-F(x)]=\infty ,\;\;\;\;\;\;p>0. \end{array}$$

Here, we suggest a new heavy-tailed distribution to provide the best description of the heavy-tailed financial data set. The new heavy-tailed distribution can obtained by using the Burr-XII (B-XII) distribution as a special case of the NG-*X* family.

Let *F*(*x*;*ξ*) = 1 − (1 + *x*^*c*^)^−*k*^, be the cdf of the two-parameter B-XII distribution, where *ξ* = (*c*, *k*). Then, a random variable say *X* is said to follow the new generalized B-XII (NGB-XII) distribution, if its cdf is given by

$$\begin{array}{c} G(x;\theta ,\xi )=1-\{\frac{{\left(1+x^{c}\right)}^{-k\theta }}{e^{1-{\left(1+x^{c}\right)}^{-k}}}\},\;\;\;\;\;\;x\geq 0. \end{array}$$

The corresponding pdf is given by

$$\begin{array}{c} G(x;\theta ,\xi )=ckx^{c-1}{\left(1+x^{c}\right)}^{-k\theta }\{\frac{\theta +{\left(1+x^{c}\right)}^{-k}}{e^{1-{\left(1+x^{c}\right)}^{-k}}}\}\;\;\;\;\;\;x>0. \end{array}$$

**Fig 16:**
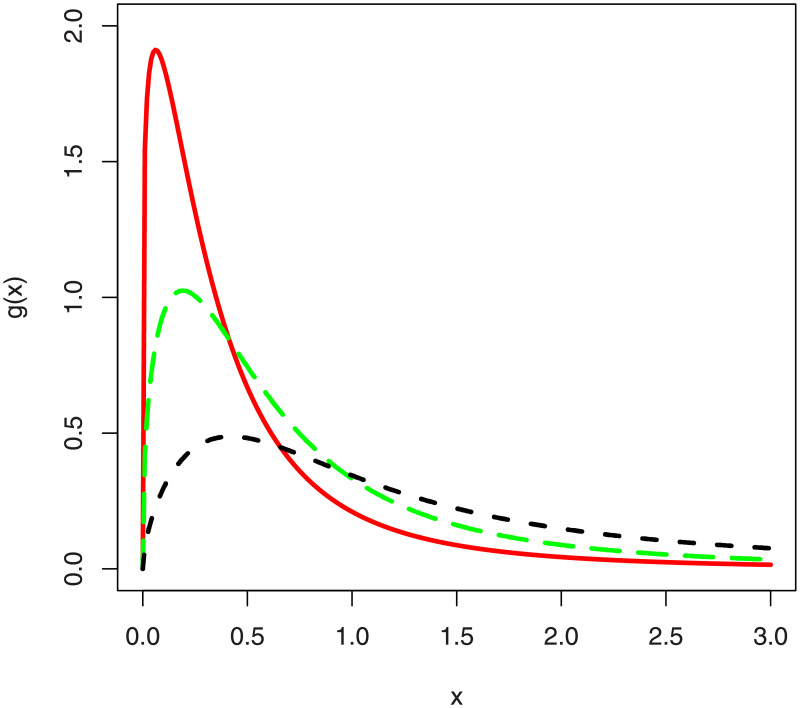
The pdf plots of the NGB-XII distribution for different values of the parameters.

Different plots for the pdf of the NGB-XII distribution are provided in Fig 16. These plots are sketched for (i) *c* = 1.2, *k* = 1.5, *θ* = 1.2 (red line), (ii) *c* = 1.4, *k* = 0.8, *θ* = 1.4 (green line), and (iii) *c* = 1.6, *k* = 0.3 and *θ* = 1.6 (black line).

From the plots provided in Fig 16, we can see that increasing the value of *c* and *θ*, the NGB-XII distribution tends to a heavy-tailed distribution. Henceforth, the NGB-XII distribution can be a good candidate distribution to model heavy-tailed financial and other related data sets. In the future, therefore, we are intended to use the proposed method to introduce new heavy-tailed distributions.

## 8 Concluding remarks

Due to the great importance of statistical distributions in modeling data in reliability engineering, we introduced and studied a new family of distributions, called a NG-*X* family. The MLEs of the model parameters along with some mathematical properties, are derived. Based on the proposed approach, a new modified form of the Weibull model called an NG-Weibull distribution is introduced and studied in detail. The NG-Weibull model is very versatile and is able to cater to the different patterns of failure rates. Due to the flexible behavior of the hf, the proposed model is capable to describe adequately the failure behavior of several lifetime datasets, particularly reliability engineering data. The usefulness of the NG-Weibull distribution is proved by analyzing the coating machine failure time data. We performed Bayesian estimation and estimated the model parameters using five different loss functions. The diagnostics measures such as the Gelman-Rubin, Geweke, and Raftery-Lewis are discussed to evaluate the MCMC procedure in the Bayesian analysis. As a future work, new models based on the proposed approach will be introduced to model complex form of reliability engineering and heavy-tailed financial data sets.
